# Endothelial Surface Glycocalyx Can Regulate Flow-Induced Nitric Oxide Production in Microvessels *In Vivo*

**DOI:** 10.1371/journal.pone.0117133

**Published:** 2015-01-09

**Authors:** Wanyi Yen, Bin Cai, Jinlin Yang, Lin Zhang, Min Zeng, John M. Tarbell, Bingmei M. Fu

**Affiliations:** Department of Biomedical Engineering, The City College of the City University of New York, New York, New York, United States of America; Emory University, UNITED STATES

## Abstract

Due to its unique location, the endothelial surface glycocalyx (ESG) at the luminal side of the microvessel wall may serve as a mechano-sensor and transducer of blood flow and thus regulate endothelial functions. To examine this role of the ESG, we used fluorescence microscopy to measure nitric oxide (NO) production in post-capillary venules and arterioles of rat mesentery under reduced (low) and normal (high) flow conditions, with and without enzyme pretreatment to remove heparan sulfate (HS) of the ESG and in the presence of an endothelial nitric oxide synthase (eNOS) inhibitor, N^G^-monomethyl-L-arginine (L-NMMA). Rats (SD, 250–300g) were anesthetized. The mesentery was gently taken out from the abdominal cavity and arranged on the surface of a glass coverslip for the measurement. An individual post-capillary venule or arteriole was cannulated and loaded for 45 min with 5 μM 4, 5-Diaminofluorescein diacetate, a membrane permeable fluorescent indictor for NO, then the NO production was measured for ~10 min under a low flow (~300 μm/s) and for ~60 min under a high flow (~1000 μm/s). In the 15 min after switching to the high flow, DAF-2-NO fluorescence intensity increased to 1.27-fold of its baseline, DAF-2-NO continuously increased under the high flow, to 1.53-fold of its baseline in 60 min. Inhibition of eNOS by 1 mM L-NMMA attenuated the flow-induced NO production to 1.13-fold in 15 min and 1.30-fold of its baseline in 60 min, respectively. In contrast, no significant increase in NO production was observed after switching to the high flow for 60 min when 1 h pretreatment with 50 mU/mL heparanase III to degrade the ESG was applied. Similar NO production was observed in arterioles under low and high flows and under eNOS inhibition. Our results suggest that ESG participates in endothelial cell mechanosensing and transduction through its heparan sulfate to activate eNOS.

## Introduction

The inner surface of blood vessels is lined with endothelial cells coated with a thin layer of endothelial surface glycocalyx (ESG). The ESG consists of proteoglycans, glycosaminoglycans (GAGs) and glycoproteins [1,2,3,4]. The GAGs in the ESG are heparan sulfate (HS), hyaluronic acid (HA), chondroitin sulfate (CS) and sialic acid (SA), of which, the most abundant one is HS, accounting for 50–90% of the GAGs [2]. Previous studies have shown that the ESG plays an important role in maintaining vessel wall permeability [5,6,7,8] and modulating circulating blood cell-vessel wall interaction [1,9,10,11,12]. Damage of ESG was found in many cardiovascular diseases, diabetes, ischemia/reperfusion, chronic infectious diseases, chronic kidney diseases [3,13,14,15,16] as well as in tumor metastasis [17]. Due to its unique location, the ESG of the microvessel wall may serve as a mechano-sensor and transducer of blood flow.

Nitric oxide (NO), the smallest signaling molecule known [18], is one of the most important protective molecules in the vasculature. Endothelial nitric oxide synthase (eNOS) is responsible for most of the vascular NO production [19,20]. NO regulates vascular tone and blood flow, inhibits platelet aggregation and adhesion, controls vascular smooth muscle proliferation and inhibits leukocyte adhesion and vascular inflammation [18,21,22]. Shear stress generated by blood flow has been demonstrated to induce NO production in coronary vasculature in dogs [23], in various sized arteries (1–8 mm diameter) of pigs [24], in small arteries of rabbits [25] and in a variety of cultured endothelial cells (ECs) [21,26,27,28]. An extensive in silico model that captures the major mechanisms of NO production in endothelial cells has been reported recently [29].

So far, at least ten candidates have been identified as mechano-sensors and transducers, including cell adhesion proteins (e.g., VE-cadherin, PECAM-1) [30,31], ion channels [32,33], tyrosine kinase receptors (e.g. vascular endothelial growth factor receptor 2) [31]; G-protein-coupled receptors and G-proteins [34], caveolae [35], primary cilia [36], actin filaments [37], nesprins [38], and integrins [39]. These structures and molecules of ECs can sense blood flow-induced mechanical stimuli and transmit them into the EC cytoplasm and nucleus to regulate vascular functions. Being the most apical structure of the ECs along with cilia facing the blood flow, the ESG may also serve as a mechanosensor and transducer for the blood flow. Florian et al. [21] found that shear induced NO production was impaired in bovine aortic endothelial cells (BAECs) when heparinase III was used to degrade HS in ESG. Depletion of HS and HA but not CS on BAECs blocks the shear-induced NO production [27]; depletion of HS, HA and CS also inhibits the shear-induced increase in hydraulic conductivity of BAEC monolayers [28]. Degradation of HS inhibits the shear-induced NO production in cultured rat aortic smooth muscle cells [40] and degradation of HA but not CS attenuates the flow-induced NO production in myotubes [41]. Mochizuki and coworkers [42] found that after hyaluronidase treatment, the shear stress-induced NO production was reduced in isolated canine femoral arteries. In an ex vivo study using porcine superficial femoral arteries, Kumagai et al [43] confirmed the role of HA in shear stress-mediated NO mechanotransduction but not HS and SA. Instead, their study implied a role of HS and SA in ROS (reactive oxygen species) regulation in the vessel wall under the shear stress stimulation. Other recent studies have shown that shear stress can alter the distribution of ESG components on the cell surface and their rates of synthesis [29] [44] [45] [46,47].

Although the ESG has been shown to regulate flow-induced NO production in arteries and in cultured arterial ECs, and Van Teeffelen et al [48] indirectly showed that heparin-impaired glycocalyx attenuates shear dependent vasodilation in arterioles of mouse cremaster muscle, there has been no direct study showing that ESG can regulate flow-induced NO production in individual microvessels. Therefore, the objective of this study was to test the hypothesis that the ESG plays a role in mechanosensing and transduction of the microvessel wall through regulating flow-induced NO production. We used fluorescence microscopy and a fluorescent NO indicator, 4, 5-Diaminofluorescein diacetate (DAF-2 DA) to measure the EC NO production [49] in individually cannulated post-capillary venules of rat mesentery under reduced (low) and normal (high) flow conditions. We have shown recently that these vessels have a substantial ESG characterized by a ~ 1 μm thick HS layer [50]. To examine the role of ESG in NO production, we used heparinase III to selectively degrade the HS of the ESG at the microvessel wall before the NO measurement under the low and high flow conditions. To further investigate the molecular mechanism by which ESG regulates the flow-induced NO production, we measured NO production in the presence of the endothelial nitric oxide synthase (eNOS) inhibitor, N^G^-monomethyl-L-arginine (L-NMMA). The results showed that heparinase treatment completely inhibited flow-induced NO production in post-capillary venules.

## Materials and Methods

### Ethics Statement

All experiments were performed on adult female Sprague–Dawley rats (250–300g), supplied by Hilltop Laboratory Animals (Scottdale, PA). All animal care and preparation procedures were approved by the Animal Care and Use Committee at the City College of the City University of New York (The protocol number is 0899).

### Animal Preparation

The methods used to prepare rat mesenteries, perfusion solutions and micropipettes for microperfusion experiments have been described in detail in [17,51,52]. A brief outline of the methods is given below with emphasis on the special features of the current experiments. At the end of experiments the animals were euthanized with excess anesthetic. The thorax was opened to ensure death.

Rats were anesthetized with pentobarbital sodium given subcutaneously at the initial dosage of 65 mg/kg followed by an additional 3 mg/dose when needed. Then the rat was transferred to a tray and kept warm at 37°C on a heating pad and monitored by a thermometer. A mid-line surgical incision (2–3 cm) was made in the abdominal wall. The mesentery was carefully taken out from the abdominal cavity and arranged on a glass coverslip to maintain circulation to the intestine and mesentery. During the entire experiment, the upper surface of the tissue was continuously superfused by a dripper with mammalian Ringer solution at ~37 ºC, which was regulated by a controlled water bath and monitored by a thermometer probe. Most microvessels chosen for the study were straight non-branched post-capillary venules, with diameters of ~25–45 μm. Another group contained arterioles of diameter ~15–35 μm. All vessels had brisk blood flow immediately before cannulation and had no marginating white cells.

### Solutions and Reagents

Mammalian Ringer solution was used for all dissections, perfusate and superfusate. The solution composition was (in mM) 132 NaCl, 4.6 KCl, 1.2 MgSO_4_, 2.0 CaCl_2_, 5.0 NaHCO_3_, 5.5 glucose, and 20 HEPES. Its pH was balanced to 7.4 by adjusting the ratio of HEPES acid to base. In addition, the perfusate into the microvessel lumen contained bovine serum albumin (BSA, Sigma) at 10 mg/ml (1% BSA-Ringer solution). 4, 5-Diaminofluorescein diacetate (DAF-2 DA), N^G^-monomethyl-L-arginine (L-NMMA), and sodium nitroprusside (SNP) were purchased from Sigma (Sigma-Aldrich, St. Louis, MO). The stock solutions of DAF-2 DA (10 mM) were prepared with 100% DMSO. The final concentrations of DAF-2 DA (5 μM), L-NMMA (1 mM) and SNP (50 mM) were achieved by dilutions of the stock with 1% BSA-Ringer solution [49]. FITC conjugated mouse anti-human heparan sulfate (Anti-HS, 10e4 epitope) was purchased from the United States Biological (Swampscott, MA). It was diluted to 1:50 (20 μg/ml) in 1% BSA-Ringer solution for labeling heparan sulfate in the microvascular endothelial surface glycocalyx. Alexa Fluor 488-labeled Griffonia (Bandeiraea) Simplicifolia Lectin II (GSL II) (Vector Labs, USA) at 40 μg/ml in 1% BSA-Ringer solution was used to recognize chondroitin sulfate. Hyaluronic acid binding protein (Millipore EMD) was first conjugated with Alexa Fluor 488 following a labeling kit from Invitrogen (Cat# A20181). Then Alexa Fluor 488-labeled hyaluronic acid binding protein at 50 μg/ml in 1% BSA-Ringer solution was used to label hyaluronic acid. A blocking solution was made of 5% goat serum (Invitrogen, Eugene, OR) in 1% BSA-Ringer. *F. heparinum* Heparinase III (50 mU/ml, IBEX, Canada) is selectively active only towards heparan sulfate (HS) [53]. All of the solutions described above were made at the time when the experiment was performed and were discarded at the end of the day.

### Intravital Microscopy

A Nikon Eclipse TE2000-E inverted fluorescent microscope was used to observe the mesentery. The tissue was observed with either transmitted white light from a light pipe suspended above the preparation or with fluorescent light from an illumination system (the monochromator with a xenon lamp FSM150Xe, Bentham Instrument Ltd., UK). The monochromator can generate the light of wavelength from 200 to 700nm. The observation of the DAF-2 labeled microvessel wall and that of fluorescently labeled glycocalyx were done by a high-performance digital 12-bit CCD camera (SensiCam QE, Cooke Corp., Romulus, MI, USA) with a Super Fluor 20x objective lens (NA = 0.75, Nikon) and recorded by InCyt Im^TM^ imaging and analyzing system (Intracellular Imaging Inc., Cincinnati, OH, USA). The excitation/emission wavelength (nm) were 485/538 nm and 490/525nm for DAF-2 and FITC, respectively.

### Determination of Perfusion Velocity in a Microvessel

Fluorescent polystyrene microspheres (3 μm diameter, Phosphorex, Inc., Hopkinton, MA) were used to measure perfusion flow velocity in a microvessel [54]. The excitation/emission wavelength for the fluorescent beads was 468/510 nm (green). The bead movement was monitored by a high performance analog 10 bit XR/MEGA-10 ICCD camera (Stanford Photonics Inc. CA) and recorded on VCR tapes. The recorded analog video images were first converted into digital movies (640×480 μm/frame at 30 frames/s under medium/low video profile) via the Microsoft media encoder (Microsoft, Seattle, WA). From the digital movies, the images of bead movement were taken by the Microsoft Live Movie Maker (Microsoft, Seattle, WA), and analyzed by NIH Image-J to determine its centerline velocity. Mean velocity in that vessel was then calculated using the correction 1/1.6 of the centerline bead velocity [55,56].

### Perfusion of a Single Microvessel under Low and High Flow Velocities

**Figure 1:**
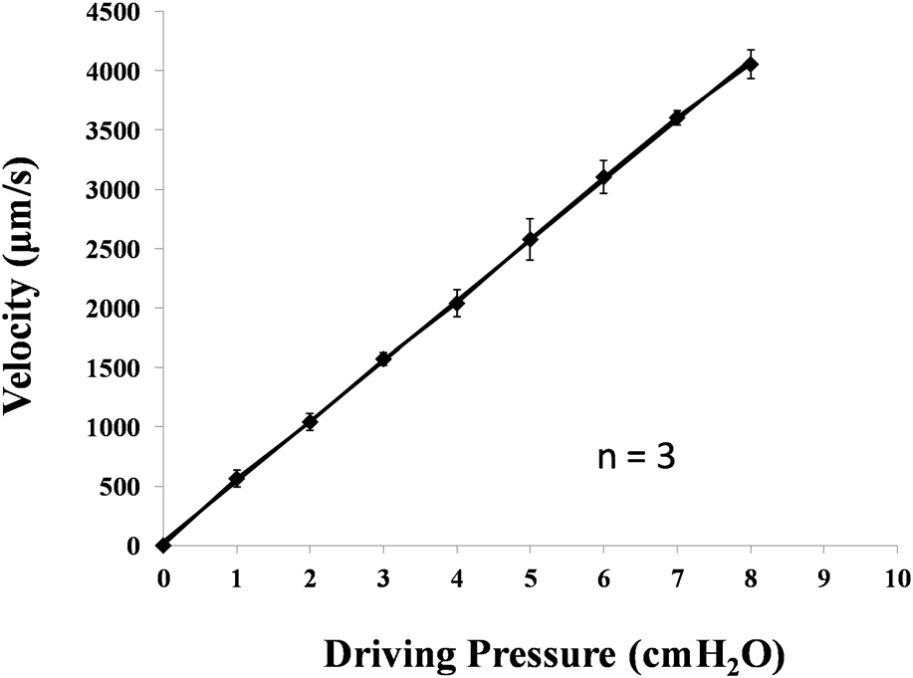
Mean perfusion flow velocity as a function of the driving pressure. The perfusion flow velocity was determined by tracking the movement of a fluorescent bead of 3 μm diameter when the vessel was cannulated and perfused at a known driving pressure measured from a water manometer. The movement of a fluorescent bead was recorded using our imaging system at various pressures for each vessel. Three vessels were tested and values shown are mean ± SE.

A single microvessel was cannulated with a glass micropipette (~15–30 μm tip diameter, World Precision Instrument Inc., Florida) and perfused with specific solutions. An initial pressure of 15–20 cmH_2_O for the post-capillary venules, or 20–30 cmH_2_O for the arterioles, was applied through the pipette to the microvessel lumen from a water manometer connected to the pipette holder. The initial pressure was set to balance the downstream blood pressure. Then the pressure was increased to a perfusion pressure. The difference between the initial pressure and the perfusion pressure was denoted as the driving pressure. The perfusion flow velocity was determined by the driving pressure and was calculated from the movement of a 3 μm fluorescent bead. The relationship between driving pressure and perfusion velocity is demonstrated in Fig. 1 for 3 vessels of typical sizes. As shown in Fig. 1, for a reduced (low) flow of ~ 300 μm/s, the driving pressure was set around 0.5 cmH_2_O; for a normal (high) flow in a post-capillary venule (~1000 μm/s) and in an arteriole (2000–2500 μm/s) [54], the driving pressure was set around ~2 cmH_2_O and 4–5 cmH_2_O, respectively.

If we assume Hagen-Poiseuille flow in a microvessel (Reynolds number is in the order of 0.01 in these mesenteric microvessels), the wall shear stress is given by (8μV_mean_)/D, where μ is the viscosity of the perfusate, which is 9.5 × 10^–3^ dyn.s/cm^2^ for 10 mg/ml BSA Ringer at 37°C [57], V_mean_ is the mean perfusion velocity (Fig. 1) and D is the vessel diameter. For the microvessels used in this study, the low flow of V_mean_ ~300 μm/s represents a mean wall shear stress of ~ 0.6 dyn/cm^2^ in a post-capillary venule and ~ 0.8 dyn/cm^2^ in an arteriole. The high flow of ~1000 μm/s represents a mean wall shear stress of ~2 dyn/cm^2^ (range1.9–2.2) in a post-capillary venule and the high flow of 2000–2500 μm/s represents a mean wall shear stress of ~6.2 dyn/cm^2^ (range 5.1–7.6) in an arteriole.

### Measurement of Endothelial NO Production in a Microvessel

The method used to measure NO production in the endothelial cells forming the microvessel wall was similar to that described in [49]. Endothelial NO levels were visualized in individually perfused microvessels using DAF-2 DA, a membrane permeable fluorescent indictor for NO, and a fluorescence imaging system. Briefly, in each experiment, a microvessel was cannulated and loaded with DAF-2 DA (5 µM) in 1% BSA-Ringer solution for 45 min at a perfusion velocity <300 μm/s. Then, focused on the mid-plane of the vessel wall, the image was first collected for 10 min at a low flow of ~300 μm/s and switched to a normal (high) flow of ~1000 μm/s for a post-capillary venule, or 2000–2500 μm/s for an arteriole by adjusting the perfusion pressure in a water manometer. The image was collected for 60 min under the high flow. To inhibit the eNOS activity, 1 mM L-NMMA was present during the loading, low and high flow periods. To degrade the ESG, the microvessel was pretreated for 1 h at a low flow with 50 mU/mL *F. heparinum* heparinase III. To test if the endothelial cells forming the microvessel wall were damaged by the enzyme treatment, the superfusate of a NO donor, sodium nitroprusside (SNP), was applied at the end of the experiment.

All images of endothelial DAF-2 were analyzed with the public domain National Institutes of Health IMAGE J program by selecting a region of interest (ROI) focused on the mid-plane of the vessel wall. ROI has a length of ~200–500 μm and a width of a vessel diameter. The baseline of the NO production (DAF-2 intensity), F_0_, was chosen as that at 45 min after DAF-2 DA loading; flow-induced temporal changes in DAF-2 intensity were expressed as F(t)/F_0_ for each vessel, where F(t) is the DAF-2 intensity at time t.

The fluorescence chemical formation of DAF-2 by NO is irreversible [58] and the detected NO-sensitive fluorescence with DAF-2 represents a cumulative production of NO. The DAF-2 fluorescence intensity vs. time curve was fit by a sigmoidal four-parameter Gompertz growth model for the NO production function f(t) [59]

$$f(t)=a\times \exp(-\exp(-\frac{t-t_{0}}{b}))+c$$ (1)

where a, b, c and t_0_ are the fitting constants, which were determined by SigmaPlot 11.2 through curve fitting the measured data. Equation 1 is an empirical formula used to describe the transient NO production under chemical stimuli [49,59,60]. From Equation 1, the NO production rate was calculated as

$$\frac{df}{dt}=\frac{a}{b}\times \exp(-(\exp(-\frac{t-t_{0}}{b})+\frac{t-t_{0}}{b}))$$ (2)

### Immuno-Labeling and Quantification of Microvessel Endothelial Surface Glycocalyx (ESG)

To quantify the ESG of the microvessel wall, FITC-conjugated HS antibody was used to label HS, the most abundant glycosaminoglycan forming the endothelial surface glycocalyx (ESG) [4,61]. Similar to our previous study [17,50], a post-capillary venule of rat mesentery was cannulated by a θ micropipette. The upper surface of the mesentery was continuously superfused by a dripper with mammalian Ringer solution at 4 ºC, which was regulated by a controlled water bath with ice and monitored using a thermometer probe. The vessel was first perfused for 15 min with a blocking solution of 5% goat serum containing 1% BSA-Ringer through one lumen of θ pipette. Then the perfusion was switched to another lumen of the pipette to inject FITC-conjugated anti-heparan sulfate (HS) in 1%BSA-Ringer (20 μg/ml) into the microvessels for ~2.5 h. The 2.5 h was long enough to allow FITC-anti-HS to infiltrate the entire depth of the ESG. After 15 min perfusion of the first perfusate to wash away the free dye, the vessel with fluorescently labeled glycocalyx (focused at the mid-plane of a vessel) was imaged by the same imaging system used in the NO measurement. The intensity of the fluorescently labeled glycocalyx in the vessel segment was measured by InCyt Im^TM^ imaging and analyzing system (Intracellular Imaging Inc., Cincinnati, OH, USA). To test the assumption that the fluorescence intensity is linearly related to the amount of the fluorescently labeled glycocalyx, we did *in vitro* calibration experiments. We used the same instrument settings in the calibration experiments as those used in the *in vivo* measurement of the fluorescently labeled glycocalyx. The linear range of FITC-anti-HS concentrations was from 0 to 50 μg/ml under our settings. We thus chose 20 μg/ml FITC-anti-HS in our experiments. We determined the amount of the fluorescently labeled glycocalyx in the vessels under control and after 1 h treatment with 50 mU/mL *F. heparinum* heparinase III, the same dosage and treatment time as for the NO measurement.

By turning on the fluorescent light under the bright field, we can observe the microvessel boundary and determine the location of FITC-anti-HS labeled ESG. We can see the FITC-anti-HS labeled ESG at the luminal side of the microvessel wall under our microscope and the fluorescent region is almost completely gone after the enzyme treatment. Since anti-HS antibody is a macromolecule of MW ~150kD and takes a couple of hours to penetrate the ESG under 4°C [50], it is very hard to cross the microvessel wall to label the matrix components at the abluminal side of the vessel wall.

To investigate if there is any CS and HA in arterioles, we used the same immunolabeling protocol with Alexa Fluor 488 conjugated GSL II and hyaluronic acid binding protein to recognize CS and HA of ESG. We did not observe either CS or HA in arterioles of rat mesentery.

### Measurement of Microvessel Diameters

Images of microvessels collected by the CCD camera were inputted into IMAGE J program and the diameter of a vessel was determined by the distance between the outer walls of the vessel. Diameters were measured at 3 locations of each vessel. The averaged value was the diameter for that vessel.

### Data Analysis

Data are presented as mean ± SE, unless indicated otherwise. Statistical analysis was performed by two-way (time and cumulative NO level) ANOVA using Sigma Plot 11.2 from Systat Software Inc. (San Jose, CA). A level of p < 0.05 was considered a significant difference in all experiments.

## Results

### Flow-Induced NO Production and Its Regulation by Endothelial Surface Glycocalyx in Post-capillary Venules

**Figure 2:**
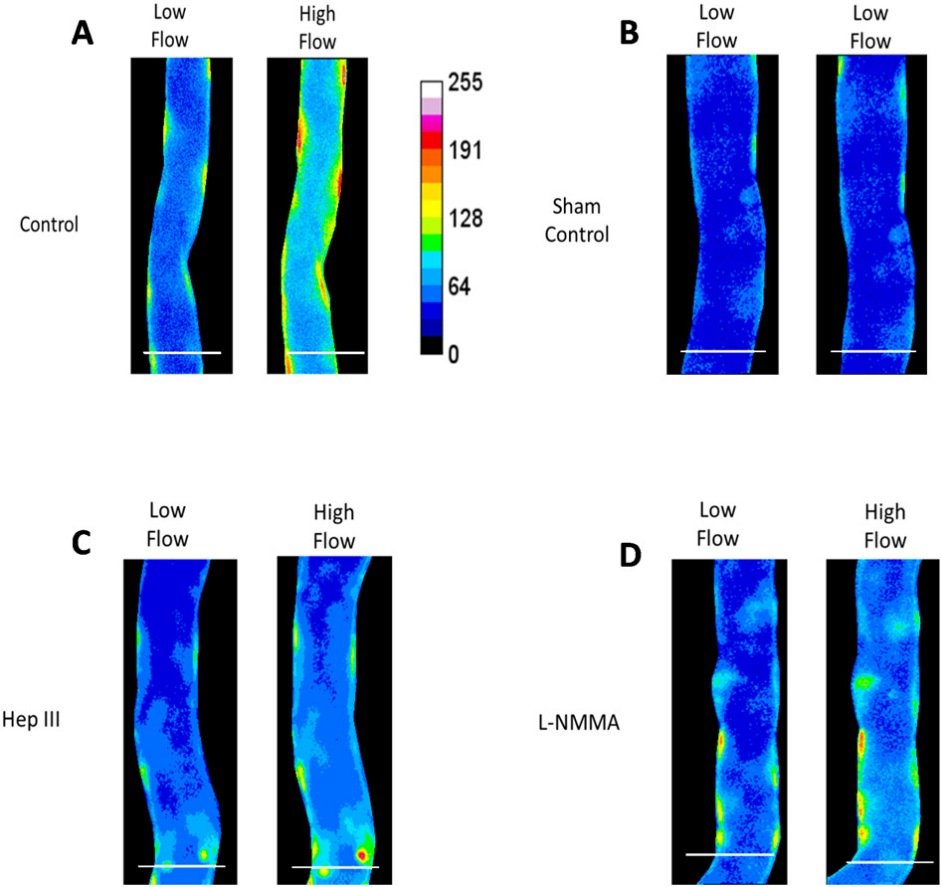
Representative DAF-2 fluorescence images for post-capillary venules. Images were taken after 10 min low flow (left panel) and an additional 60 min high flow (right panel). A) control (1% BSA Ringer); B) sham control (low flow over entire time); **C)** 1 h pretreatment of heparinase III; and **D)** in the presence of L-NMMA. Scale bar is 50 μm.

**Figure 3:**
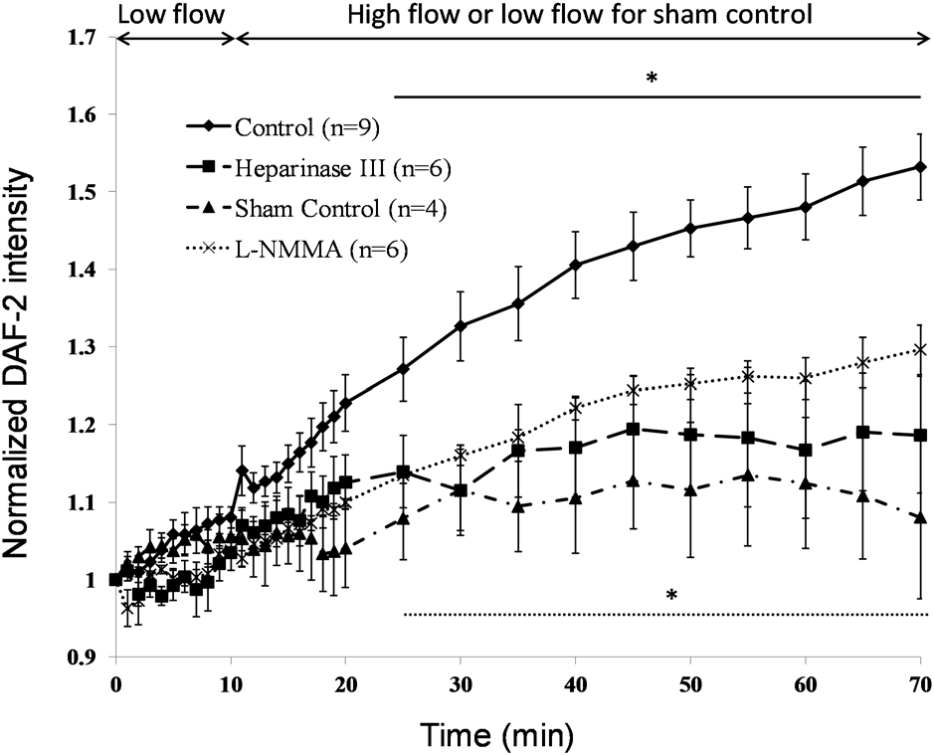
Flow-induced increases in NO production (DAF-2 intensity normalized by that after 45min DAF-2 DA loading) under various conditions in post-capillary venules. The solid line with diamonds is for the control perfusing 1% BSA-Ringer under the low flow (~300 μm /s) for 10 min and the high flow (~1000 μm/s) for 60 min; the dash-dot-dash line with triangles is for the sham control perfusing 1% BSA-Ringer under the low flow for 70 min; the dashed line with squares is for that with 1 h pretreatment of heparinase III; and the dotted line with crosses is for that in the presence of L-NMMA. * p < 0.05 compared with that at 10 min low flow (for the control and L-NMMA treatment).

Fig. 2 demonstrates typical DAF-2 images of post-capillary venules under various conditions. The left Fig. in each panel shows the image at 10 min after low flow and the right one shows that the image at 60 min after high flow. Fig. 3 plots the normalized DAF-2 fluorescence intensity, F(t)/F_0_, under the low (~300 μm/s) and high (~1000 μm/s) perfusion velocities. The baseline intensity F_0_ is that after 45 min DAF-2 DA loading for each vessel (t = 0 in Fig. 3). The solid line with diamonds is for the control with the perfusate of 1% BSA Ringer; the dashed line with squares is for the 1 h pretreatment of heparinase III; the dotted line with crosses is for that in the presence of an eNOS inhibitor, L-NMMA, and the dash-dot-dash line with triangles is for the sham control under low flow only. We can see from Fig. 3, 10 min low flow insignificantly increased the NO-DAF-2 by less than 5% under all the conditions (p > 0.05). After switching to the high flow, NO-DAF-2 was not significantly increased until 15 min later for the control and for that in the presence of L-NMMA (p <0.03). After 15 min high flow, NO-DAF-2 increased to 1.27 ± 0.04-fold of its baseline, NO continuously increased under the high flow, reaching a plateau in ~50 min, and to 1.53 ± 0.04-fold in 60 min (n = 9). Inhibition of eNOS by 1 mM L-NMMA attenuated the flow-induced NO increase to 1.13 ± 0.01-fold in 15 min (p = 0.018 compared to the control) and 1.30 ± 0.03-fold in 60 min (p < 0.001 compared to the control, n = 6), respectively.

In contrast, the flow-induced NO production was almost completely abolished by the 1 h pretreatment with 50 mU/mL heparinase III (n = 6) (p > 0.07). To examine if the enzyme treatment damaged the endothelial cells and to verify that the endothelial cells in each vessel were well loaded with DAF-2, at the end of the 60 min high flow, a NO donor, sodium nitroprusside (SNP), was applied to the superfusate and a large sudden increase in the NO-DAF-2 fluorescence intensity was observed in each vessel (data not shown). If endothelial cells are damaged, the loaded DAF-2 in their cytoplasm would be out and washed away by the perfusate, adding the NO donor, SNP, would not induce the fluorescence in individual endothelial cells forming the vessel wall. Therefore superfusion of SNP is widely used to test if endothelial cells forming the microvessel wall are damaged by the treatment [49,60]. Prior permeability study also reported that 1 h treatment with 50 mU/mL heparinase III did not change other components of the microvessel wall except degrading the ESG [17].

**Figure 4:**
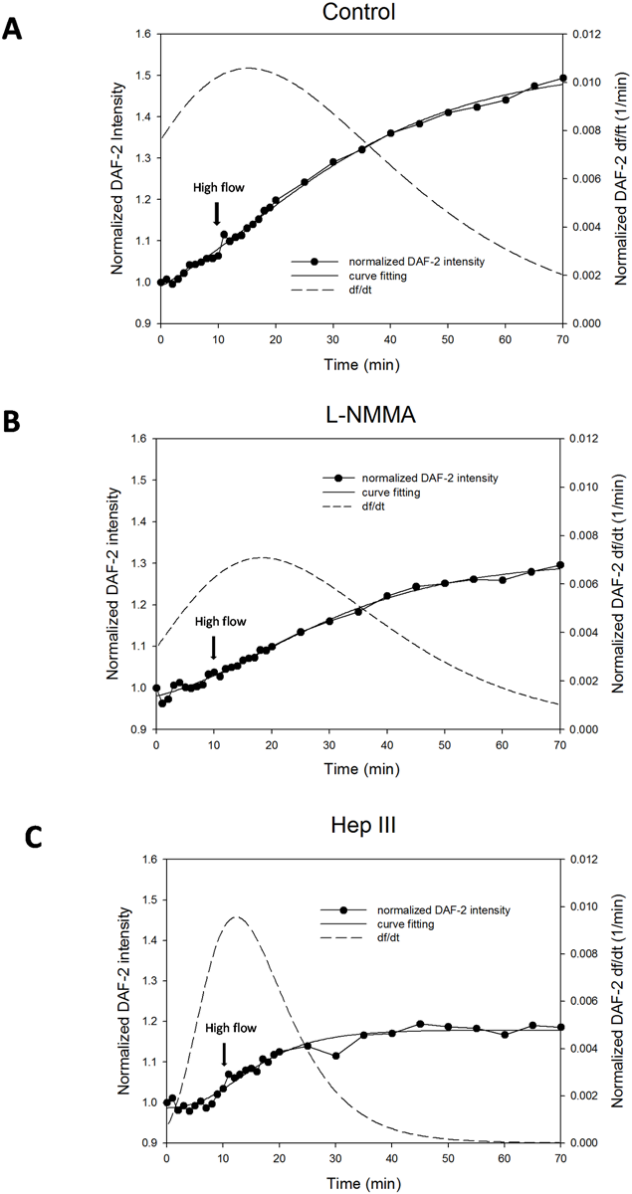
Curve fitting for the flow-induced increases in NO production (DAF-2 intensity normalized by that after 45min DAF-2 DA loading) (smooth solid line) and production rate (df/dt) (dashed line) in post-capillary venules. A) control (1% BSA Ringer); B) in the presence of L-NMMA; and C) 1 h pretreatment of heparinase III. The filled circles are the measured data.

After curve fitting using Equation 1 for the normalized DAF-2 intensity, F(t)/F_0_, we obtained the normalized NO production function f(t). Its derivative, Equation 2, gives the NO production rate df/dt. In Fig. 4, we plotted both NO production (the symbols for the measured data and the solid line for the fitting curve) and the production rate (dashed line). This sigmoidal four-parameter Gompertz growth model fit very well for the flow-induced NO production data with R^2^ > 0.97 for all the cases except for the sham control when there was no NO generated. Fig. 4A is for the control case by perfusing 1%BSA Ringer under low and high flows. Different from the sudden and transient increase in the NO production by chemical stimuli such as bradykinin [59] and platelet-activating factor (PAF) [60], the flow-induced NO production was gradual and the highest NO production rate occurred at about 5 min after switching to the high flow, which was ~0.01/min. After the peak, the endothelial cells continued to produce NO at a slower rate. Inhibition of eNOS by L-NMMA attenuated the NO production, reduced the production rate but did not change the temporal pattern of the NO production by the flow (Fig. 4B). On the contrary, enzymatic degradation of ESG altered the NO production pattern by the flow. Fig. 4C shows that after 1h pretreatment of heparinase III, the flow-induced NO production increase was sudden and transient, similar to that observed by applying bradykinin [59] and PAF [60]. Interestingly, the peak production rate after the enzyme treatment, ~0.01/min, was the same as that without enzyme treatment.

### Heparan Sulfate ESG Removal by Heparinase III

**Figure 5:**
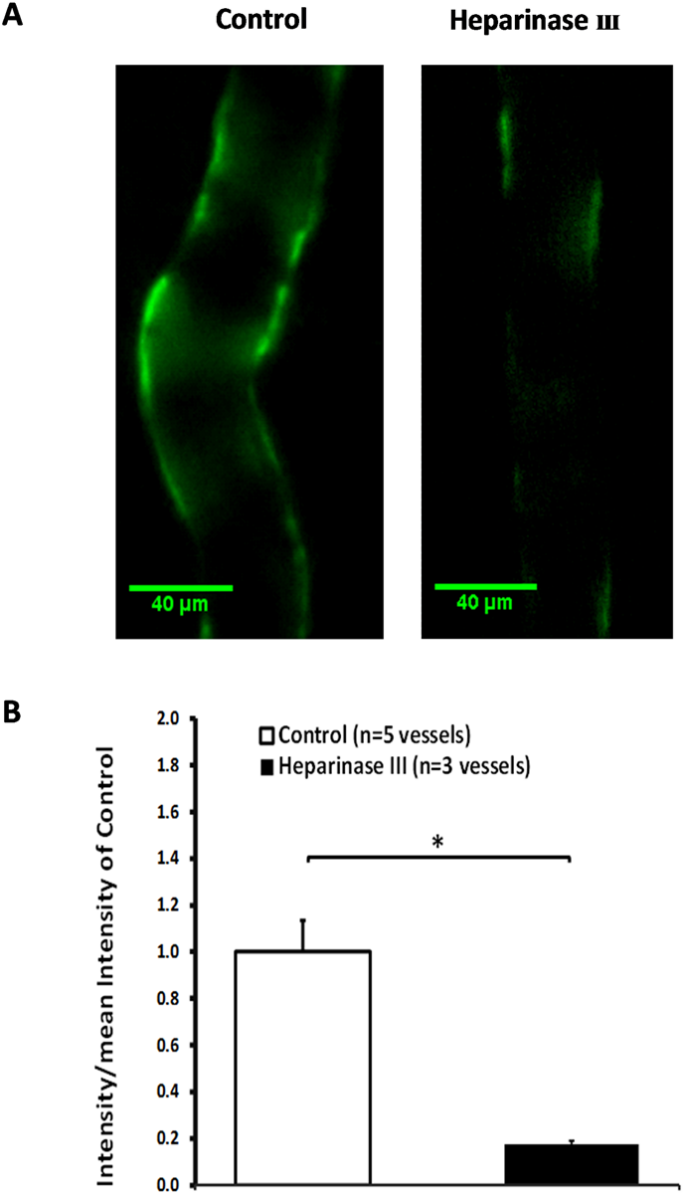
Images of fluorescently labeled heparan sulfate in a control vessel (left in Fig. 5A) and a vessel treated with heparinase III for 1 h (right in Fig. 5A). Fig. 5B shows the comparison of the intensity of the fluorescently labeled heparan sulfate in 5 control vessels and that in 3 heparinase III treated vessels. * p < 0.001.

To examine the removal of the ESG, we did the immunostaining of heparan sulfate (HS) before and after the enzyme treatment in individual post-capillary venules. Fig. 5 indicates that 1 h 50 mU/mL heparinase III treatment removed more than 80% of the ESG (p < 0.001).

### Flow-Induced NO Production in Arterioles

**Figure 6:**
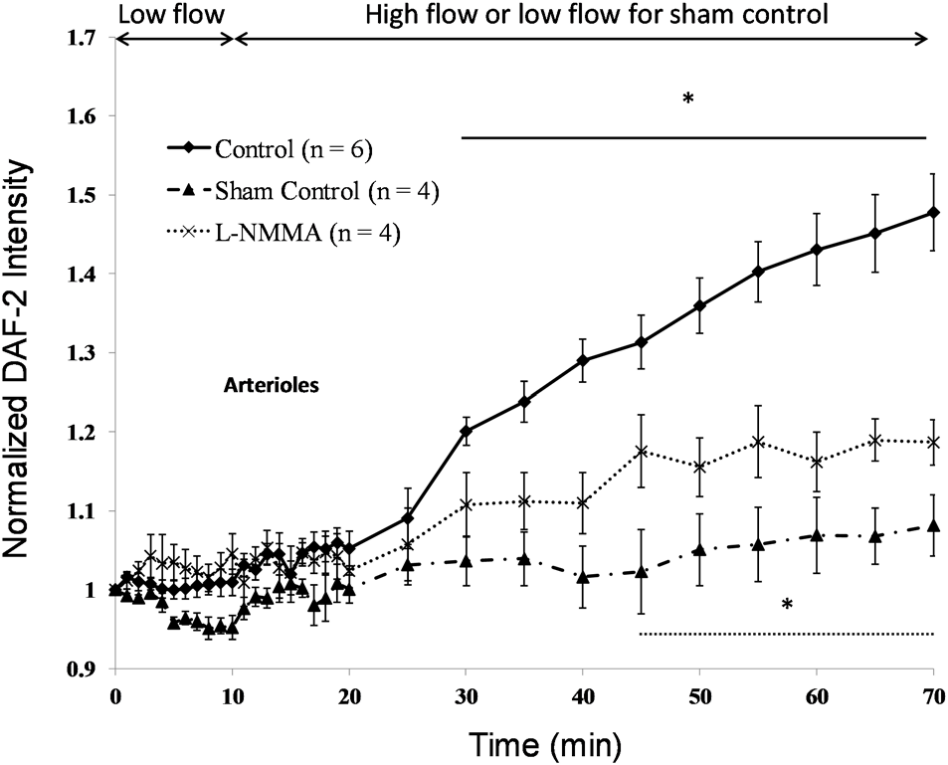
Flow-induced increases in NO production (DAF-2 intensity normalized by that after 45min DAF-2 DA loading) under various conditions in arterioles. The solid line with diamonds is for the control of perfusing 1% BSA-Ringer under the low flow (~300 μm/s) for 10 min and the high flow (~2000–2500 μm/s) for 60 min; the dash-dot-dash line with triangles is for the sham control of perfusing 1% BSA-Ringer under the low flow for 70 min; and the dotted line with crosses is for that in the presence of L-NMMA. * p < 0.05 compared with that at 10 min low flow (for the control and L-NMMA treatment).

In our previous study [50], we could not observe significant HS in arterioles although we found significant HS in capillaries and post-capillary venules of rat mesentery. In the current study, we also performed immunolabeling of chondroitin sulfate (CS) and hyaluronic acid (HA) in the arterioles. No significant CS or HA was found in arterioles. To examine if the flow can also induce NO production in mesenteric arterioles in the absence of ESG, we measured NO production in arterioles. Fig. 6 demonstrates the results, which are similar to those observed in post-capillary venules. We raised the high flow perfusion velocity to ~2000–2500 μm/s, which is the mean blood flow velocity in mesenteric arterioles [54]. After switching to the high flow, NO-DAF-2 was not significantly increased until 20 min later for the control and until 35 min later for that in the presence of L-NMMA (p <0.05). After 20 min high flow, NO-DAF-2 increased to 1.20 ± 0.02-fold of its baseline, NO continuously increased under the high flow, reached a plateau in ~50 min, and to 1.48 ± 0.05-fold in 60 min. Inhibition of eNOS by 1 mM L-NMMA attenuated the flow-induced NO increase to 1.19 ± 0.03-fold (p < 0.001) in 60 min.

**Figure 7:**
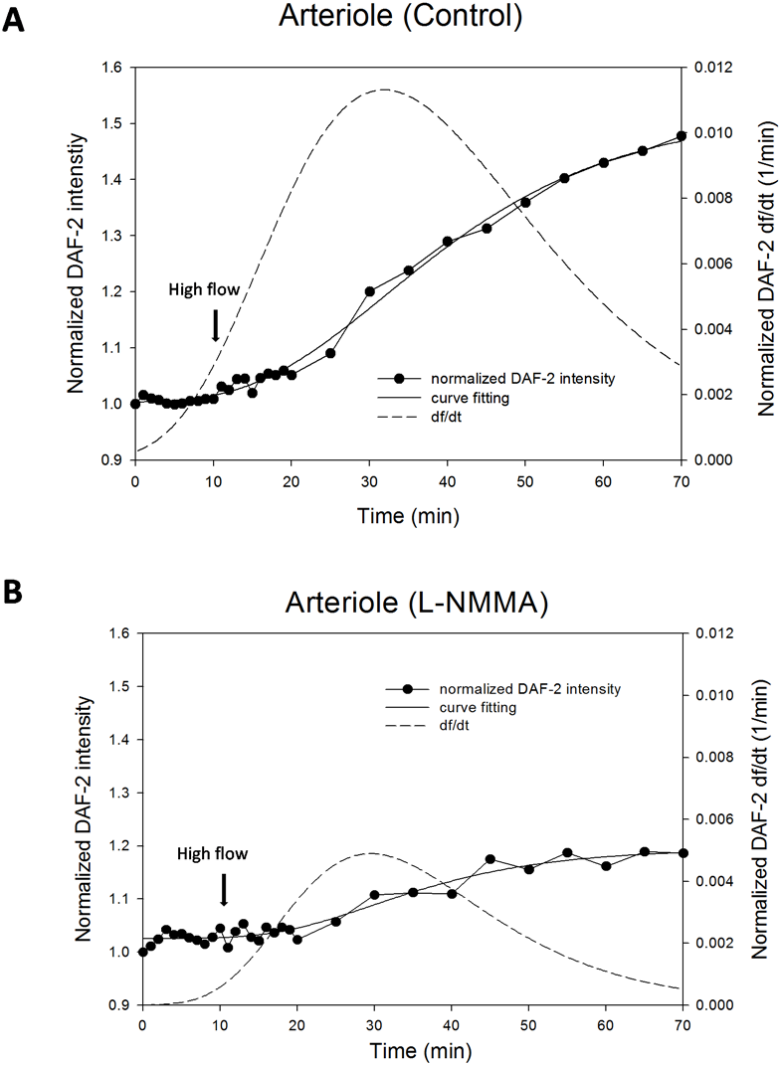
Flow-induced increases in NO production (DAF-2 intensity normalized by that after 45min DAF-2 DA loading) and production rate (df/dt) in arterioles A) control (1% BSA Ringer); B) in the presence of L-NMMA. The filled circles are the measured data, the solid line is the fitting curve and the dashed line is the production rate.

In parallel with the post-capillary venules, in Fig. 7, we plotted both NO production (the symbols for the measured data and the solid line for the fitting curve) and the production rate (dashed line). The sigmoidal four-parameter Gompertz growth model also fitted very well for the flow-induced NO production data in arterioles with R^2^ > 0.95. Fig. 7A is for the control case by perfusing 1%BSA Ringer under low and high flows and Fig. 7B for that in the presence of L-NMMA. Similar to that in the post-capillary venules, the flow-induced NO production was gradual and the highest NO production rate occurred at about 20 min after switching to the high flow, which was ~0.01/min. After the peak, the endothelial cells continued to produce NO at a slower rate. Inhibition of eNOS by L-NMMA attenuated the NO production, reduced the production rate but did not change the temporal pattern of the NO production by the flow.

### Flow-Induced NO Production Does Not Change the Diameter of Microvessels

As a vasodilator, NO induced by flow may increase the size of microvessels. We examined the vessel diameters right after 45 min DAF-2 DA loading, at 10 min low flow and at 60 min high flow. Tables 1,2 show that the mean diameters of both post capillary venules and arterioles have no significant changes under all conditions in our experiments (p > 0.7).

**Table 1:** Diameters of post-capillary venules under low (~300 μm/s) and high (~1000 μm/s) flows.

	**0 min Low Flow**	**10 min Low Flow**	**60 min High Flow**
Control (n = 9)	37.01 ± 2.71	37.47 ± 2.86	39.56 ± 2.71
Heparinase III (n = 6)	34.70 ± 3.41	34.76 ± 3.41	34.68 ± 3.24
L-NMMA (n = 6)	32.97 ± 1.38	32.87 ± 1.40	33.15 ± 1.39
Sham control (n = 4)	34.48 ± 3.67	34.20 ± 3.70	34.62 ± 3.60 (low flow)

Data shown are Mean ± SE (μm). p > 0.7.

**Table 2:** Diameters of arterioles under low (~300 μm/s) and high (~2000–2500 μm/s) flows.

	**0 min Low Flow**	**10 min Low Flow**	**60 min High Flow**
Control (n = 6)	25.47 ± 3.26	26.53 ± 3.39	27.68 ± 3.48
L-NMMA (n = 4)	29.64 ± 2.24	29.81 ± 4.29	31.52 ± 1.51
Sham control (n = 4)	26.24 ± 1.81	27.18 ± 1.71	27.16 ± 2.21 (low flow)

Data shown are Mean ± SE (μm). p > 0.7.

## Discussion

Fluorescent images of DAF-2-loaded microvessels provide a direct visualization and quantification approach for analyzing the spatial and temporal NO production in ECs of intact microvessels [49,60]. Cannulation and perfusion of a single microvessel enable us to control the vessel flow rate properly [17,52]. In vivo perfusion of enzyme and immunostaining of the ESG in an individual microvessel enable us to more precisely degrade and quantify the specific ESG component [17,50]. By using these recently developed techniques in our and other labs, we demonstrated in the current study that degradation of the ESG at the post-capillary venule, specifically the HS component of the ESG, inhibited the flow-induced NO production in the ECs forming the microvessel wall, suggesting that the ESG plays a major role in mechano-sensing and transducing in microvessels. This is consistent with previous studies in cultured EC monolayers [21,27,28] and in arteries [42,43].

When exposing human umbilical vein endothelial cells (HUVECs) to steady laminar flow, Kuchan and Frangos [26] observed a biphasic response in NO production, with an initial burst of NO production within minutes followed by a gradual NO release over hours. It was also demonstrated that the initial rapid NO release was G protein and Ca^2+^ dependent but the later slower response was G protein and Ca^2+^ independent and shear level dependent [26]. A similar biphasic response of NO production was observed in BAECs when exposed to step changes in shear stress [21,62]. An NOS inhibitor, N^G^-amino-L-arginine (L-NAA), completely blocked the flow-mediated NO release in HUVECs [26]. In the intact post-capillary venules, we did not observe a rapid NO production when switching to the high flow (Fig. 3). Instead, the NO production was rather gradual, though at a higher production rate in the beginning after switching to the high flow, which peaked around 5 min after the onset of high flows (Fig. 4). Enzymatic degradation of the HS completely inhibited the flow-generated NO production in intact post-capillary venules (Fig. 3,4c). In the presence of eNOS inhibitor, L-NMMA, the NO production in response to the flow was attenuated significantly (Figs. 3,4), suggesting that the flow-induced NO production is through activation of eNOS. L-NMMA is a relatively non-selective inhibitor of all NOS isoforms and is claimed to be a potent eNOS inhibitor. The current results showed that enzymatic degradation of ESG was a better inhibitor of the flow-induced NO production by endothelial cells.

The molecular mechanisms by which ESG regulates flow-induced NO production in the microvessel wall are not yet known. One possibility is a glypican-caveolae-eNOS mechanism. The transmembrane syndecans and the membrane bound glypicans are the major core protein families of heparan sulfate proteoglycans found on the EC plasma membrane [2,63]. Glypicans, to which HS binds, are linked to caveolae where eNOS resides [2]. When flow imposes drag force on HS, the mechanical stimuli would be transmitted via the glypican to the caveolae and trigger the NO production by eNOS inside the caveolae. This has been demonstrated recently in BAECs where it was shown that glypican-1 not syndecan-1 is the proteoglycan core protein mediating eNOS activation by shear stress [64].

Prior studies showed that 30 min treatment with 60 mU/ml heparinase III removed 60% of the HS in the ESG of BAEC monolayers [47] and 10 min treatment with 50 mU/ml heparinase III only reduced the ESG thickness by 57% in the post-capillary venule of rat mesentery [65]. Our current study revealed that 1 h treatment with 50 mU/ml heparinase III removed more than 80% of the HS in post-capillary venules (Fig. 5). Previous studies using the same type of enzyme reported no other changes in the structural components of the microvessel wall except for degrading the ESG [17]. No reaction of the endothelial cells or any significant off target degradation of CS or HA in cultured cell monolayers were reported even at much higher doses [66]. Since HS is the dominant GAG of the EC glycocalyx [2], our enzyme treatment should degrade most of the ESG. Without the ESG, flow-induced mechanical stimuli such as shear stress can directly act on the EC plasma membrane. Although the cumulative NO production in the microvessel wall was not significantly increased by enhancing the perfusion flow rate after degradation of the ESG (Fig. 3), when switching to the high flow, the NO production rate increased immediately, similar to what has been observed by applying agonists, such as bradykinin in intact endothelium of coronary arteries [59] and PAF in intact post-capillary venules [60]. In BAECs, addition of bradykinin induced significant NO production that was not inhibited by pretreatment with heparinase III [21]. These results suggest that additional mechanisms in the absence of the ESG can also trigger NO production, by the potential mechano-sensors and transducers at the EC plasma membrane and within the EC cytoskeleton, e.g., cell adhesion molecules, G-protein-coupled receptors and G-proteins, actin filaments and integrins [4,34].

We previously observed a FITC-anti-HS labeled ESG layer of ~ 1 μm in capillaries and post-capillary venules of rat mesentery and a ESG layer of ~2 μm at rat aorta, but we did not observe significant HS in arterioles [50], and neither HA or CS in the current study. Without finding significant GAGs, we still observed flow-induced NO production in arterioles, and the flow-induced NO production was also attenuated by an eNOS inhibitor, L-NMMA (Fig. 6). These observations are consistent with findings of Williams [57] in frog mesenteric capillaries. She reported that pronase treatment, which completely degraded the glycocalyx, led to an enhanced sensitivity of endothelial hydraulic conductivity to shear stress that has been shown in other studies to be mediated by enhanced NO production [67]. This is also consistent with a study by Lopez-Quintero et al. [28] that showed that the sensitivity of NO production to shear stress was not inhibited when the glycocalyx was highly disrupted in protein-free media. In our study, when the ECs forming the arteriole wall are not covered by significant ESG, the flow-induced mechanical stimuli can directly act on the EC plasma membrane. Then other EC mechano-sensors and transducers may come into play to regulate vascular functions [4,34]. Additional studies will be required to unravel this puzzle and elucidate the functional mechanisms of ESG and various EC mechano-sensors and transducers mediating NO production in arterioles.

As a vasodilator, the flow-induced NO slightly increased the diameter of post-capillary venules and arterioles under control conditions without enzyme treatment and eNOS inhibition although the increase was not significant (p > 0.7, Tables 1,2). One reason is that we set up the high flow rate as the normal blood flow rate in respective microvessels, which does not require the reduction in the flow resistance by increasing the vessel size. Although the temporal patterns in the ESG mediated flow-induced NO production are different from those in agonist (PAF)-mediated NO production [60] in the same type of microvessels, the NO production vs. time curve by both factors satisfies the sigmoidal four-parameter Gompertz growth model, suggesting a common NO generation mechanism by which ECs respond to external stimuli. Further investigation is required to elucidate this common mechanism.
